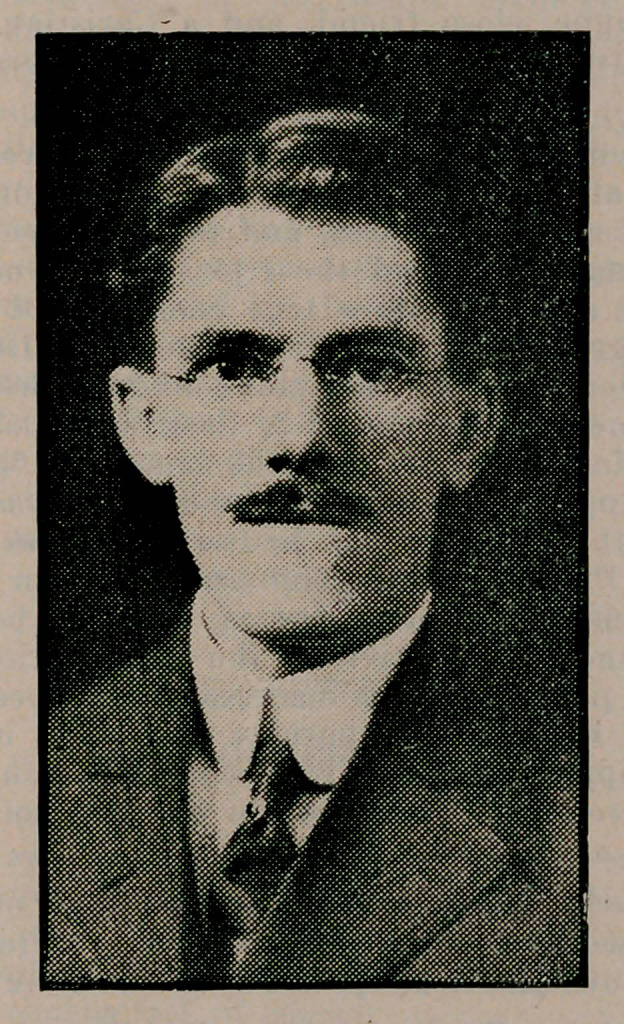# A Memorial to William Irving Thornton

**Published:** 1916-04

**Authors:** 


### A Memorial to William Irving Thornton.

The officers and members of The Practitioner's Club having learned with deep regret of the death of Dr. William Irving Thornton on January 16, 1916, at this the first meeting following his death, desire to give expression to their sorrow in the loss of an honored colleague, and of their high appreciation of Dr. Thornton as a man, and a physician and surgeon. Dr. Thornton had an attractive personalty. He was cheerful always, possessing a sweetness of disposition, and as one approached him and looked into his face his eyes at once were full of thought and kindness, and as one entered into conversation with Dr. Thornton or listened to his papers and discussions before medical bodies, one was impressed that simple straightforward honesty of purpose and sincerity of thought were marked attributes of his nature. Though unassuming and retiring by nature Dr. Thornton was energetic and optimistic in his work. He was a skilled diagnostician, careful, conscientious, persevering, untiring, resourceful in his care of the sick: always kind, sympathetic, and beloved alike by his patients and associates.

Looking back over the whole seventeen years of Dr. Thornton's medical practice there has been no blot, no departure from the practice of high ideals and high standards.

He was steadfast in the employment only of the most highly approved scientific instruments and methods of procedure in his diagnostic and therapeutic work. And for this we, his associates in the medical profession, honor and cherish his memory, and recognize in his death the loss of an honored colleague, close friend, and a Christian gentleman, fully appreciating that we shall miss his encouragment and helpful advice.

Also, the members of this club desire to express their unbounded sympathy to the stricken ones of the home to which he brought so much happiness, and to his immediate associates, and lovingly commend them to the care of the Great Shepherd who alone can give true comfort and consolation in this their great hour of extreme sorrow. Dr. Thornton was a charter member of this club; he was present at the organization meeting November 2, 1905, and took an active part. He made the motion that the temporary chairman of the meeting appoint a committee, and later was appointed a member of the committee on by-laws. He has been Vice-President and President of the club and chairman of the committees on membership and program. He has been faithful in his attendance, and active in the presentation of cases, specimens and papers, and his discussions have been frequent, broad-minded, helpful contributions. In view of which it seems highly appropriate that we should adopt this memorial to serve as a record of our pride in our late colleague, and of our sorrow at his loss.

Therefore, be it Resolved, that a copy of this our heartfelt tribute to his worth be engrossed and conveyed to the family of our late fellow, and it be spread upon our minutes.

Committee—John R. Gray, John Middleton, Edward L. Frost.

Members—C. E. Curtice, F. A. Drake, H. K. DeGroat, T. F. Dwyer, John R. Gray, E. L. Frost, William F. Jacobs, John Middleton, D. C. McKenney, Edward A. Sharp, James Stoddart, William H. Thornton, Geo. A. Himmelsbach, James E. King, Chester D. Moses, H. C. Rooth, George A. Sloan, W. G. Taylor, W. Irving Thornton, Harry M. Meed.

## Figures and Tables

**Figure f1:**